# Exploring the Interactions Between RHAU Peptide and G-Quadruplex Dimers Based on Chromatographic Retention Behaviors

**DOI:** 10.3390/molecules29245915

**Published:** 2024-12-14

**Authors:** Ju Wang, Jun-Qin Qiao, Chao Liang, Xue-Wen Guo, Meng-Ying Zhang, Wei-Juan Zheng, Hong-Zhen Lian

**Affiliations:** 1 State Key Laboratory of Analytical Chemistry for Life Science, School of Chemistry & Chemical Engineering and Center of Materials Analysis, Nanjing University, 163 Xianlin Avenue, Nanjing 210023, China; wangju1128@163.com (J.W.); guoxuewen@nju.edu.cn (X.-W.G.); 2 Nanjing Zhulu Pharmaceutical Technology Co., Ltd., 28 Kexin Road, Nanjing 211500, China; lc24168@sina.com; 3 State Key Laboratory of Pharmaceutical Biotechnology, School of Life Sciences, Nanjing University, 163 Xianlin Avenue, Nanjing 210023, China; wjzheng@nju.edu.cn

**Keywords:** size-exclusion chromatography, SEC, G-quadruplex dimers, G4 dimer–RHAU interaction, retention behavior

## Abstract

G-quadruplex (G4), an important secondary structure of nucleic acids, is polymorphic in structure. G4 monomers can associate with each other to form multimers, which show better application performance than monomers in some aspects. G4 dimers, the simplest and most widespread multimeric structures, are often used as a representative for studying multimers. RHAU, a G4 ligand, has been reported to recognize G4 dimers. However, there are few reports focusing on interactions between RHAU and different G4 dimers. In this work, interactions between RHAU peptide and six G4 dimers were investigated by size-exclusion chromatography (SEC). It was revealed that compared to the hybrid G4 monomer, the hybrid tandem unstacked G4 dimer could form special binding sites, leading to a weak interaction with RHAU. It was also found that the steric hindrance at terminal G-tetrads of a special Z-G4 structure greatly weakened their interactions with RHAU. Additionally, RHAU exhibited stronger interactions with intermolecular stacked/interlocked parallel dimers than with intramolecular tandem stacked parallel dimers. This work enriches the understanding of interactions between RHAU and G4 dimers, which is conducive to the elucidation of G4 polymorphism, and provides a strong reference for studying G4 multimer–peptide interactions.

## 1. Introduction

G-quadruplex is polymorphic in structure and is affected by various factors [1]. G4 monomers can associate with each other to form dimers, trimers, and more advanced structures, which is called the multimerization of G4s [2]. Due to differences in the functions of diverse G4 structures, increasingly more attention has been paid to the study of specific G4 structures. In recent years, researchers have found that G4 multimers exhibit better performance than G4 monomers in some properties, such as better enzyme catalytic ability [3,4,5], highly sensitive detection of various targets [6,7,8,9], and significant enhancement on fluorescent response of probes [10]. G4 multimers also possess important biological functions, for instance, regulation of gene expression [2]. For these merits, G4 multimers have attracted researchers’ increasing interest. Some G4 sequences exist mainly in the form of dimers and show the physiological effect of inhibiting HIV integrase activity [11,12]. Furthermore, when experimental conditions change, G4 sequences can transform from monomers to dimers [13,14]. Therefore, the acquisition of G4 dimers is more convenient than that of other types of multimers. There are three types of G4 dimers. One is a stacked dimer formed by two monomers stacking on each other via the π–π stacking of terminal G-tetrads. The second is a tandem dimer in which two G4 monomers are formed simultaneously at different positions within the same nucleic acid sequence and are linked by one or more bases (“beads in a string” or tandem dimers). On the basis of these two dimers, G4 multimers can be formed by further stacking of monomers or by lengthening of the nucleic acid sequence of a tandem dimer, which are the most common routes of producing G4 multimers. In addition, different dimerization modes can be combined. For example, with respect to tandem dimers, they can be either stacked or unstacked. The third type of dimer is an interlocked dimer in which a guanine from one G4-forming sequence is paired with three guanines from another sequence to form a G-tetrad plane, thus forming the interlocked dimer. On account of the extensive existence and easy acquisition of G4 dimers, they are an ideal representative for studying G4 multimers.

Ligands that are able to specifically recognize G4s have great potential in disease treatment and biological process regulation, facilitating the development of novel ligands and the study of G4–ligand interactions. Although most G4-specific ligands reported so far are focused on recognizing G4 monomers [15,16,17,18,19,20,21,22,23,24,25], ligands targeting multimers have been gradually developed [26,27,28,29,30]. RHAU (M_w_ = 6484.3), an RNA helicase associated with AU-rich elements, has the potential to target G4s [31,32]. RHAU plays a significant role in telomerase regulation [33], heart development [34], and gene expression [35]. Most biological functions of RHAU are mediated through G4s, indicating the important biological value of understanding interactions between RHAU and G4s. It has been reported that RHAU peptide can bind to G4 dimers via end stacking [36]. However, there are few reports focusing on interactions between RHAU and different types of G4 dimers. Based on the important biological role of RHAU, and the great potential and research value of G4 multimers, it is believed that interactions between RHAU and different G4 dimers are worthy of extensive exploration.

In this work, we investigated the interactions of RHAU peptide (subsequently referred to as RHAU) with six G4 dimers by size-exclusion chromatography (SEC), circular dichroism (CD), ultraviolet–visible absorption spectroscopy (UV–Vis), and native polyacrylamide gel electrophoresis (native-PAGE). It was found that the hybrid tandem unstacked G4 dimer is able to form special sites for RHAU binding. Additionally, the steric hindrance at terminal G-tetrads had a significant effect on RHAU binding to a Z-G4 dimer, as indicated by the weak interaction between them. Moreover, it was revealed that RHAU shows a stronger interaction with the intermolecular stacked/interlocked parallel dimer than with the intramolecular tandem stacked parallel dimer. Investigations in this work help to expand the applications of the biological functions of RHAU and elucidate G4 polymorphism. At the same time, taking G4 dimers as a starting point, the findings provide a strong reference for the study of G4 multimer–ligand interactions and for the development of ligands specifically targeting G4 multimers.

## 2. Results and Discussion

### 2.1. CD and UV–Vis Analysis

**Figure 1:**
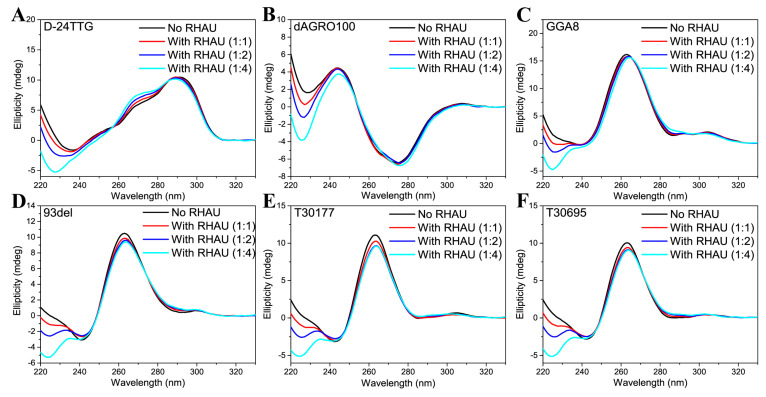
CD spectra of G4 dimers without and with RHAU at different molar ratios: (**A**) D-24TTG, (**B**) dAGRO100, (**C**) GGA8, (**D**) 93del, (**E**) T30177, and (**F**) T30695. The G4 concentration was fixed at 10 μM, and the molar ratios of G4:RHAU were 1:0 (black lines), 1:1 (red lines), 1:2 (blue lines), and 1:4 (cyan lines).

Six G4-forming sequences that were able to form dimers were selected. Firstly, CD measurements were performed. As presented in Figure 1, all sequences showed characteristic CD signals of G4 structures, indicating the successful formation of G4s. D-24TTG displayed two positive peaks at 267 nm and 295 nm and a negative peak at 238 nm, representing the formation of a hybrid G4 structure [27,37]. GGA8, 93del, T30177, and T30695 all had positive peaks at 265 nm and negative peaks at 245 nm, indicating that they all formed parallel G4 structures [11,12,38,39]. It is noteworthy that the CD spectrum of dAGRO100 did not match the characteristic CD signals of three common G4 conformations (parallel, anti-parallel, and hybrid). Instead, it showed a positive peak at 245 nm and a distinct negative peak at 275 nm. According to previous reports, these characteristic CD peaks were attributed to a left-hand G4 structure (Z-G4) [40,41].

In order to understand the interactions between RHAU and various G4 dimers, CD measurements were also performed on G4+RHAU mixtures at different ratios. In our previous work [42], it was reported that RHAU has no CD signals from 220 nm to 330 nm because of the negligible absorption of Trp and Tyr residues in RHAU [43]. Therefore, differences in CD signals observed before and after RHAU addition were entirely assigned to structural changes in G4 dimers induced by RHAU. For D-24TTG (Figure 1A), as the amount of RHAU increased, its positive peaks at 267 nm and 295 nm showed a slight blue-shift along with an enhanced signal at 267 nm. This phenomenon indicated that the parallel elements of hybrid D-24TTG gradually increased. It is reported that when ligands induce hybrid D-24TTG G4 to transform to parallel structures, its positive peak at 267 nm is evidently enhanced, the positive peak at 295 nm is weakened, and the negative peak at 238 nm is also weakened, accompanied by a position shift to 242 nm [27]. However, in this work, although the CD signals of D-24TTG changed to some extent after the addition of RHAU, these changes were quite small and the negative peak at 238 nm did not move to a higher wavelength but moved to a lower wavelength. These differences demonstrated that D-24TTG here did not transform into a parallel conformation and was still a hybrid conformation. With regard to dAGRO100 and GGA8, as exhibited in Figure 1B,C, only when the ratio of G4:RHAU reached 1:4 were their signals at 275 nm and 265 nm weakened slightly and red-shifted, meaning that RHAU had a weak effect on these two dimers. With respect to 93del, T30177, and T30695, as shown in Figure 1D–F, RHAU had a slight impact on their CD signals. Specifically, when the G4:RHAU ratio changed from 1:0 to 1:2, the signals at 265 nm of these three dimers gradually decreased, with no new signals appearing at other wavelengths, pointing out that RHAU only affected G-tetrad stacking without causing structural transformation. When the ratio changed from 1:2 to 1:4, there were no further changes in the CD signals of these three G4s, meaning that their structures reached a stable state at a 1:2 ratio. Further comparison demonstrated that in the presence of RHAU, changes in the CD signals of 93del and T30695 were similar to each other, while the signal intensity of T30177 decreased more evidently. Differences in the attenuation amplitudes of the characteristic peaks of each G4 dimer reflected distinct interactions between RHAU and different G4 dimers, even though no apparent changes in CD signals were observed for all G4 dimers in the presence of RHAU, emphasizing that the main structure of each dimer was maintained, which was beneficial for the analysis of subsequent experimental results.

Furthermore, the melting temperature (*T*_m_) of the studied G4 dimers was determined in the absence and presence of RHAU by monitoring the ellipticity at the characteristic wavelength of each dimer. As shown in Figure S1 and Table S1, the *T*_m_ of D-24TTG was 49.3 °C, suggesting a relatively low thermal stability compared with that of other G4 dimers. Upon the addition of RHAU, a decreased *T*_m_ of 3.8 °C was observed, indicating that RHAU had a weak destabilizing effect on D-24TTG. Compared to D-24TTG, Z-G4 dAGRO100 exhibited a higher *T*_m_ of 53.9 °C, suggesting better thermal stability. Both GGA8 and T30177 showed high thermal stability, with *T*_m_ values of 100.8 °C and 82.9 °C, respectively. Obviously, RHAU stabilized dAGRO100, GGA8, and T30177 for the increased *T*_m_. With regard to 93del and T30695, they were too stable to obtain the *T*_m_ value within the temperature range we monitored. On the whole, all the studied parallel G4 dimers had a relatively high thermal stability, and the effects of RHAU on the stability of G4 dimers were different.

**Figure 2:**
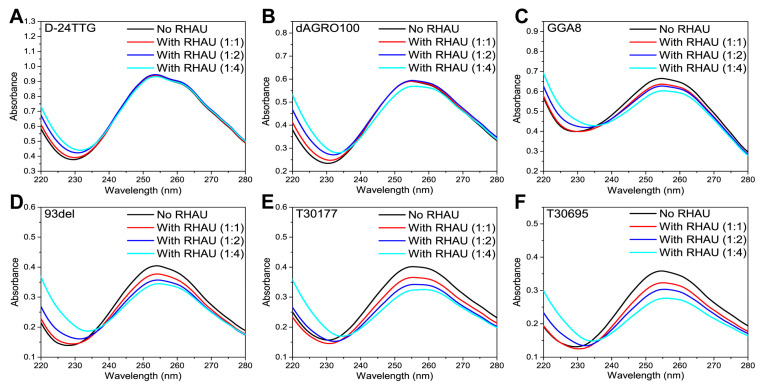
UV–Vis spectra of G4 dimers without and with RHAU at different molar ratios. (**A**) D-24TTG; (**B**) dAGRO100; (**C**) GGA8; (**D**) 93del; (**E**) T30177; (**F**) T30695. G4 concentration was fixed at 2 μM, and the molar ratios of G4:RHAU were 1:0 (black lines), 1:1 (red lines), 1:2 (blue lines), and 1:4 (cyan lines).

We also carried out UV–Vis detections on G4+RHAU mixtures at different ratios (Figure 2) to understand the interactions between RHAU and these G4 dimers. Studies show that DNAs have the maximum absorption at 260 nm, and changes in DNA structures lead to hypochromicity and red shifts of UV–Vis spectra [44]. Therefore, UV–Vis analysis can also be used to examine the influence of external factors on nucleic acid structures. In our previous work, RHAU only had UV–Vis absorption below 240 nm [42], so the observed changes in UV absorbance at 260 nm were entirely attributable to structural changes in G4s induced by RHAU. As presented in Figure 2A, RHAU showed a negligible effect on the UV–Vis spectrum of D-24TTG at any G4:RHAU ratio, which meant that RHAU showed no apparent interference in the structure of D-24TTG, consistent with CD results. For dAGRO100 (Figure 2B), only when the G4:RHAU ratio was 1:4 did its UV–Vis absorbance decrease obviously, in agreement with the CD results in Figure 1B. A slight hypochromic effect occurred after RHAU addition, but no position shift of signals was observed, indicating that the influence of RHAU on the dAGRO100 G4 structure was reflected in the enhancement of the stacking of G-tetrads [45]. As illustrated in Figure 2C–F, compared to dAGRO100, the UV–Vis absorptions of GGA8, 93del, T30177, and T30695 displayed a more apparent hypochromic effect with an increase in RHAU, pointing out that RHAU caused more enhancement of the stacking of G-tetrads of these four dimers. Differences in the hypochromic effects observed for each dimer revealed that RHAU had different impacts on the G-tetrad stacking of these six G4 dimers.

### 2.2. SEC Analysis

**Figure 3:**
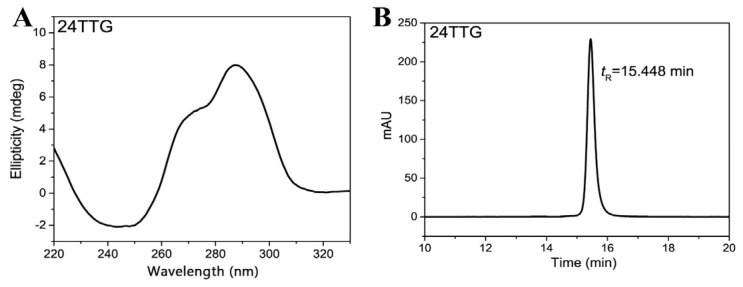
CD spectrum (**A**) and SEC chromatogram (**B**) of 24TTG. Chromatograms were obtained under isocratic mode at a rate of 0.5 mL min^−1^ on a TSKgel G2000SWXL column (7.8 × 300 mm i.d.) with 50 mM KH_2_PO_4_ as the mobile phase (pH 7.0). The concentration of 24TTG was 10 μM. The column temperature was maintained at 30 °C, and the detection wavelength was 260 nm.

Subsequently, SEC analysis was performed on these G4 dimers and their G4+RHAU mixtures. 24TTG, which only formed a G4 monomer, was selected, and its retention time (t_R_) and molecular weight (M_w_) were used as a reference to better analyze and assign chromatographic peaks for each studied dimer. Annealed 24TTG folded into a hybrid G4 structure (Figure 3A), in line with a previous report [46]. 24TTG was separated and analyzed under the same chromatographic conditions as all the dimers. As illustrated in Figure 3B, 24TTG (M_w_ of a single strand = 7563.97) had only one chromatographic peak, with a t_R_ of 15.448 min, corresponding to the hybrid G4 monomer.

**Figure 4:**
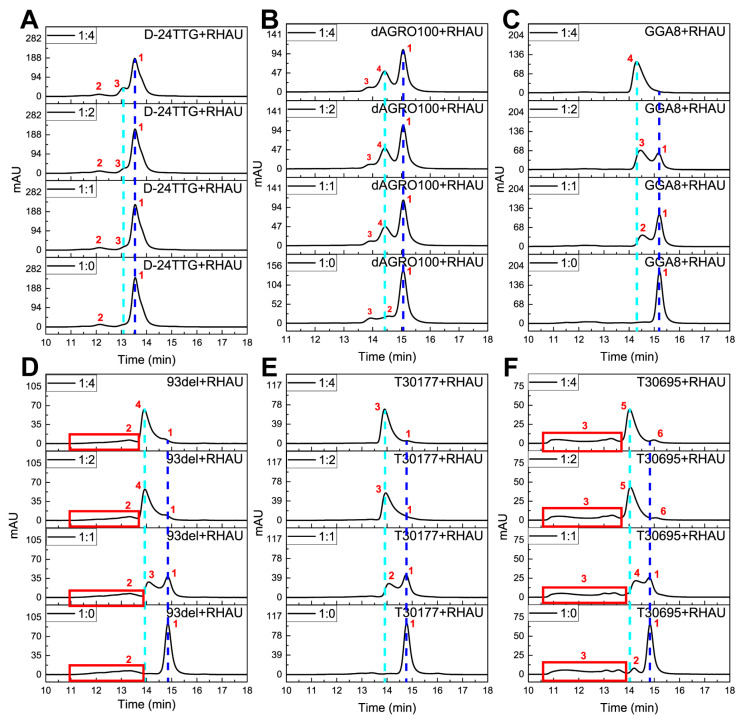
SEC chromatograms of G4 dimers with RHAU at different molar ratios: (**A**) D-24TTG, (**B**) dAGRO100, (**C**) GGA8, (**D**) 93del, (**E**) T30177, and (**F**) T30695. Ratios: 1:0, 1:1, 1:2, and 1:4. Chromatograms were obtained under isocratic mode at a rate of 0.5 mL min^−1^ on a TSK_gel_ G2000SW_XL_ column (7.8 × 300 mm i.d.) with 50 mM KH_2_PO_4_ (pH 7.0) as the mobile phase. The concentration of G4s was 10 μM. The column temperature was maintained at 30 °C, and the detection wavelength was 260 nm.

SEC results of the six dimers and their G4+RHAU mixtures are exhibited in Figure 4. With regard to D-24TTG (Figure 4A, M_w_ of a single strand = 14,682.53), without RHAU, there existed a main peak 1 at 13.553 min. Since the M_w_ of D-24TTG was twice that of 24TTG, and the hybrid conformation of D-24TTG was not capable of efficient π–π stacking, peak 1 was assigned to an intramolecular tandem dimer consisting of two unstacked hybrid G4 monomers [27,37]. With the addition of RHAU (M_w_ = 6484.3), a small peak 3 (t_R_ = 13.225 min) appeared adjacent to peak 1, which belonged to the dimer–RHAU complex, indicating that a weak interaction occurred between RHAU and D-24TTG. As the amount of RHAU increased, peak 3 was slightly enhanced, representing an increase in the amount of the D-24TTG–RHAU complex. Structurally, the base composition of the G4 monomer in the D-24TTG dimer is the same as that of 24TTG. In our previous work [42], no interactions were observed between hybrid 24TTG and RHAU, even when the ratio of G4:RHAU was 1:4. Hence, the weak interaction occurring between RHAU and D-24TTG suggested that the hybrid tandem unstacked dimer had some special properties compared to its corresponding hybrid G4 monomer, which provided additional sites for the recognition of RHAU. To investigate the binding mechanism between RHAU and D-24TTG, we used Hdock Server [47] (http://hdock.phys.hust.edu.cn/ accessed on 25 November 2024) to perform molecular docking simulation. In contrast, it was found that there was no binding between RHAU and 24TTG (Figure S2A), which was consistent with the results of SEC [42]. With regard to D-24TTG, the results demonstrated that RHAU bound laterally to the outer side of the G4 dimer (Figure S2B). This might be attributed to the TTA base in the loop extending the dimer in the vertical direction, providing a spatial basis for the binding of RHAU and D-24TTG. However, this particular combination represents an unconventional binding method. So, the binding strength is relatively weaker than that in classic binding models, such as stacking, groove, and intercalation bindings. In Figure 4A, there was a small peak 2 (t_R_ = 12.145 min), whose signal strength and peak position remained unchanged at any G4:RHAU ratio. The sequence composition of D-24TTG determined that it was impossible to form a multimer of a higher molecular weight. Therefore, it was speculated that peak 2 was derived from an incompletely folded structure of the D-24TTG sequence, whose spatial structure was less compact than that of a completely folded tandem dimer, thus resulting in weaker retention. Meanwhile, this incompletely folded structure had no special sites for RHAU binding, so it had no interactions with RHAU, which explained why its retention was not affected by RHAU addition. Compared to the other five dimers, the t_R_ of D-24TTG was much shorter. This was because D-24TTG had a large molecular weight and the two G4 monomers of the D-24TTG dimer were unstacked, leading to a less compact structure than that of stacked and interlocked dimers. Both these reasons made the D-24TTG dimer larger in size and thus weaker in SEC retention, which also indicated that in SEC, both the molecular weight and the spatial compactness of an analyte play an important role in retention.

For dAGRO100 (M_w_ of a single strand = 8934.77), without RHAU, a main peak 1 appeared at 15.060 min (Figure 4B). The M_w_ of dAGRO100 was larger than that of 24TTG, and it formed an intramolecular tandem dimer in which two Z-G4 monomers stacked on each other and each monomer contained two G-tetrad layers [40]. Therefore, dAGRO100 showed weaker retention than 24TTG, and peak 1 was attributed to this tandem stacked left-handed G4 dimer. After the addition of RHAU, peak 4 (t_R_ = 14.435 min) with weaker retention emerged, which corresponded to the dimer–RHAU complex, indicating that an interaction occurred between dAGRO100 and RHAU. With increasing addition of RHAU, peak 4 was enhanced, representing an increase in the amount of the dAGRO100–RHAU complex. Further analysis revealed that when the G4:RHAU ratio changed from 1:1 to 1:2, the enhancement in the intensity of peak 4 was smaller than that when the ratio changed from 1:0 to 1:1, and the intensity of peak 4 remained unchanged when the G4:RHAU ratio changed from 1:2 to 1:4. Such a change trend indicated that the interaction between dAGRO100 and RHAU was saturated at 1:1. The residual peak 1 showed that even at a high concentration of RHAU, a large amount of free dimers was still left, pointing out that the interaction between dAGRO100 and RHAU was weak. A characteristic of a Z-G4 structure is that some of its thymine bases collapse onto the terminal G-tetrad instead of turning outward, and hydrogen bonds are formed between the O4’ atoms of these residues and the guanine amino protons of adjacent G-tetrads [40,41]. RHAU binds to G4s primarily by stacking on terminal G-tetrads [48]. Therefore, these collapsing thymine bases and the resulting hydrogen bonds in the Z-G4 structure increase the steric hindrance at terminal G-tetrads, hindering RHAU from stacking on them. In addition to peak 1, there also existed two small peaks in Figure 4B at 1:0, i.e., peak 2 (t_R_ = 14.552 min) and peak 3 (t_R_ = 13.946 min). At a 1:2 ratio, peak 2 disappeared, while peak 3 remained unchanged. Based on t_R_ and changes in the retention behavior, it was inferred that peak 2 belonged to a tandem unstacked dimer and peak 3 corresponded to an incompletely folded structure of dAGRO100. The molecular size of a tandem unstacked dimer was slightly larger than that of a tandem stacked dimer, so the t_R_ of peak 2 was a little smaller than that of peak 1. The molecular size of an incompletely folded structure was apparently larger than that of a completely folded dimer; thus, the t_R_ of peak 3 was evidently smaller than that of peak 1 and peak 2. RHAU drove the unstacked dimers to transform to stacked ones and further to form a G4–RHAU complex (peak 4), so peak 2 disappeared after RHAU addition and turned to peak 4. RHAU did not have the ability to induce a single-stranded nucleic acid to form G4 structures, so peak 3 was not affected by the addition of RHAU, which was similar to the change in peak 2 of D-24TTG that was also assigned to an incompletely folded structure.

With respect to parallel GGA8 (M_w_ of a single strand = 7790.05, Figure 4C), its molecular weight was similar to that of 24TTG, and it also formed an intramolecular tandem dimer in which two parallel G4 monomers stacked on each other and each G4 monomer consisted of two G-tetrad layers [38]. In the absence of RHAU, the dominant peak 1 (t_R_ = 15.207 min) in Figure 4C belonged to the intramolecular tandem parallel G4 dimer. After the addition of RHAU, peak 1 was markedly weakened, and a new peak 2 with weaker retention (t_R_ = 14.525 min), corresponding to the GGA8–RHAU complex, emerged, demonstrating that an obvious interaction occurred between GGA8 and RHAU. As more RHAU was added, peak 2 was gradually enhanced, which meant that more dimer–RHAU complex was produced. When the G4:RHAU ratio changed, a slight shift in the position of peak 2 was also observed, i.e., peak 2 to peak 3 (1:2) and peak 3 to peak 4 (1:4), reflecting that the GGA8–RHAU complex underwent a structural change and eventually reached a stable state. When the ratio was 1:4, peak 1 disappeared, and only peak 4 existed, which indicated that all GGA8 dimers were combined with RHAU to form a complex in this case. Although dAGRO100 and GGA8 were both tandem stacked dimers, dAGRO100 exhibited weaker interactions with RHAU, which was because the large steric hindrance at terminal G-tetrads mentioned earlier in dAGRO100 (Z-G4) greatly weakened its interactions with RHAU.

The remaining three parallel dimers, 93del, T30177, and T30695, exhibited similar retention behaviors at different G4:RHAU ratios. In the absence of RHAU, the dominant peak 1 of 93del (M_w_ of a single strand = 5202.35, t_R_ = 14.850 min; Figure 4D) corresponded to its interlocked parallel G4 dimer [11]. Meanwhile, the main peaks 1 for T30177 (M_w_ of a single strand = 5448.53, t_R_ = 14.777 min;, Figure 4E) and T30695 (M_w_ of a single strand = 5184.33, t_R_ = 14.829 min; Figure 4F) belonged to their respective intermolecular stacked dimers, which were formed by two independent parallel G4 monomers stacking on each other [12,39]. The M_w_ of a single strand of 93del was higher than that of T30695; however, 93del had stronger retention than T30695. This was because an intermolecular interlocked dimer (93del) was more compact than an intermolecular stacked dimer (T30695), resulting in a larger t_R_ of 93del. In addition to peak 1, 93del also had a wide peak at 11.0–13.8 min (peak 2), which was presumed to be substances of higher molecular weights. Although both T30177 and T30695 were intermolecular stacked dimers, T30177 had only one obvious peak 1 without RHAU, while T30695 also had a small peak 2 (t_R_ = 14.197 min) and a wide peak 3 (t_R_ = 11.0–14.1 min). According to t_R_, peak 2 of T30695 was deduced to be a G4 trimer, and peak 3 was assigned to multimers of higher molecular weights. The reason for such difference between T30177 and T30695 was likely to be due to the bulge in the monomer of T30177 (the position marked in red in Table 1), which was close to the terminal G-tetrad and hindered the further stacking of the T30177 monomer to form high-ordered multimers.

On the addition of RHAU, peaks 1 of 93del, T30177, and T30695 were all evidently weakened, while new peaks with weaker retention appeared, indicating that these three dimers all had strong interactions with RHAU. When the G4:RHAU ratio was 1:1, there emerged peak 2, peak 3, or peak 4 with weaker retention (t_R_ = 14.086 min for 93del, t_R_ = 14.075 min for T30177, t_R_ = 14.272 min for T30695), all of which corresponded to respective dimer–RHAU complexes. When the ratio changed to 1:2, peak 1 decreased to a small size (93del and T30177) or even disappeared (T30695), while the peaks of dimer–RHAU complexes increased markedly. Moreover, when the G4:RHAU ratio changed from 1:1 to 1:2, small position shifts of dimer–RHAU peaks were observed for all three dimers (peak 3 to peak 4 for 93del, peak 2 to peak 3 for T30177, peak 4 to peak 5 for T30695). This indicated to us that the structures of these dimer–RHAU complexes became more stable and reached an equilibrium state at a G4:RHAU ratio of 1:2. Differently, a stronger-retained peak 6 emerged for T30695 at 1:2, which was presumed to be an impurity and could not be detected when the content of peak 1 was high owing to the overlap. Differences in the amount of residual free dimers (peaks 1) reflected different levels in interactions between various dimers and RHAU. With regard to 93del and T30695, when the amount of RHAU continued to increase (1:4), the peaks of dimer–RHAU complexes did not further change, suggesting that their binding to RHAU reached saturation at a 1:2 ratio. However, for T30177, when the G4:RHAU ratio changed from 1:2 to 1:4, the peak shape of peak 3 further improved, emphasizing that interactions between T30177 and RHAU did not reach a fully stable state at a 1:2 ratio.

The intensity of peak 1 at different G4:RHAU ratios reflected that GGA8 had a much larger amount of remaining free dimers at a 1:2 ratio than 93del, T30177, and T30695, indicating much weaker interactions between GGA8 and RHAU. Moreover, the structure of the GGA8–RHAU complex reached a stable state until the G4:RHAU ratio reached 1:4, which also supported the weak interaction between GGA8 and RHAU. Differences in the retention behaviors of GGA8, 93del, T30177, and T30695 dimers pointed out that RHAU had stronger interactions with parallel intermolecular interlocked/stacked dimers than with parallel intramolecular tandem stacked dimers. Additionally, RHAU is reported to prefer to bind to the 5′-terminal G-tetrad of G4 because the slightly prominent sugar-phosphate portion of the 3′-terminal causes a greater steric hindrance, and RHAU binds to the 3′-terminal G-tetrad only at a high G4 concentration or only when 5′-5′ stacked dimers exist [49]. 93del, T30177, and T30695 are all 5′-5′ stacked dimers, while dAGRO100 is a 3′-5′ stacked dimer, so it seems that RHAU is more inclined to bind to dAGRO100. However, the interaction between dAGRO100 and RHAU was, in fact, much weaker because the large terminal steric hindrance in the dAGRO100 dimer greatly weakened the binding of RHAU. This demonstrated that the steric hindrance at terminal G-tetrad was the main factor affecting interactions between RHAU and G4 dimers.

Combining the results of CD, UV–Vis, and SEC, the chromatographic peaks of each dimer in Figure 4 were carefully assigned and are summarized in Table 2.

### 2.3. Native-PAGE Analysis

**Figure 5:**
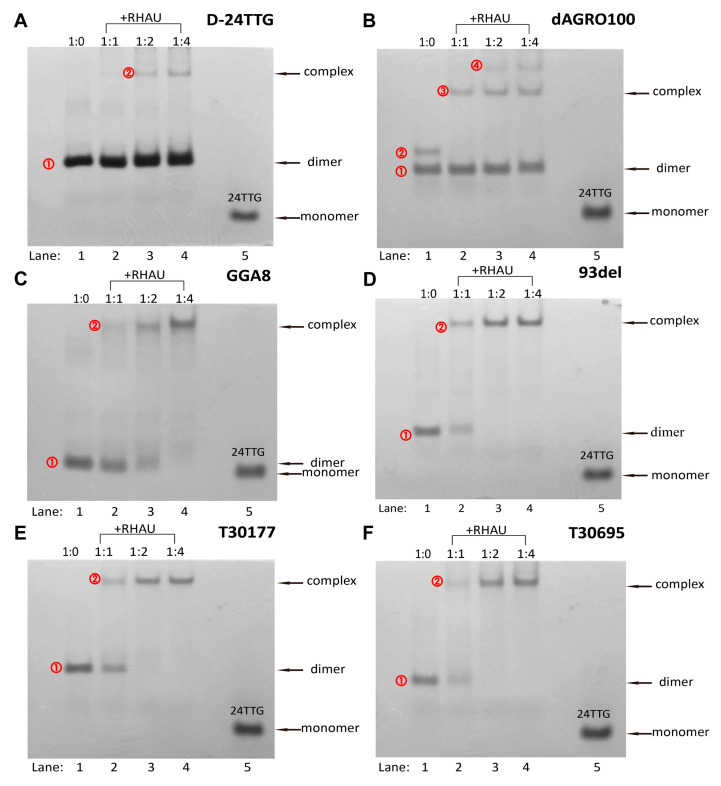
Native-PAGE images of different G4 dimers without and with RHAU at different molar ratios: (**A**) D-24TTG, (**B**) dAGRO100, (**C**) GGA8, (**D**) 93del, (**E**) T30177, and (**F**) T30695. Lanes 1–4: the molar ratios of G4:RHAU were 1:0, 1:1, 1:2, and 1:4.

In order to better understand the interactions between RHAU and each dimer, and to verify the reliability of SEC results, these dimers and their corresponding G4+RHAU mixtures were analyzed by native-PAGE. Similarly, 24TTG was used as a reference [40]. As presented in Figure 5, 24TTG had only one migrating band corresponding to its hybrid G4 monomer, and this band was taken as a reference for the G4 monomer. On the whole, in the absence of RHAU, except for dAGRO100, the remaining five dimers all showed only one band ① each, with a slower migration than that of 24TTG, each band belonging to its respective dimer, consistent with expectations. It was seen that the position of band ① of GGA8 is close to that of 24TTG. This was because GGA8 and 24TTG had similar molecular weights and the dimer of GGA8 was an intramolecular tandem dimer, resulting in similar migrating. Band ① of dAGRO100 also corresponded to its dimer, and a slower-migrating band ② appearing simultaneously with band ① was deduced to be a tandem unstacked dimer, corresponding to peak 2 in Figure 4B.

As illustrated in Figure 5A, when the amount of RHAU was low (1:1), band ① of the D-24TTG dimer remained unchanged, indicating that D-24TTG was not affected by RHAU. When the ratio of G4:RHAU reached 1:2, a new light-colored and narrow band, band ②, with slower migration appeared, representing the formation of a small amount of the G4–RHAU complex. This phenomenon was in agreement with the results in Figure 4A and indicated a weak binding between D-24TTG and RHAU. Differently, a chromatographic peak belonging to the dimer–RHAU complex emerged when the G4:RHAU ratio was 1:1 in SEC (Figure 4A), while the migrating band ② of the dimer–RHAU complex was detected at a 1:2 ratio in electrophoresis. This was ascribed to the reason that the amount of the D-24TTG–RHAU complex was too small to be effectively visualized in electrophoresis at a ratio of 1:1, which reflected the advantage of chromatography in detecting substances of small amounts. It was found in both SEC results (Figure 4A) and electrophoresis results (Figure 5A) that D-24TTG weakly interacted with RHAU. However, in our previous work [42], 24TTG had no interactions with RHAU, which once again verified that compared to the corresponding hybrid G4 monomer, hybrid tandem unstacked G4 dimers were capable of forming some special sites for ligand recognition.

With regard to the intramolecular tandem stacked dimer dAGRO100 (Figure 5B), with the addition of RHAU, the change in the color of band ① was not obvious, meaning that the interaction between RHAU and dAGRO100 is weak, in accordance with SEC results in Figure 4B. When the G4:RHAU ratio was 1:1, a slow-migrating band ③ corresponding to the dimer–RHAU complex appeared, while band ② disappeared, which was in accord with the appearance of peak 4 and the disappearance of peak 2 in Figure 4B. When the G4:RHAU ratio changed from 1:1 to 1:2, the color of band ③deepened and did not further change at a 1:4 ratio, consistent with the change trend of the signal intensity of peak 4 in Figure 4B. Unexpectedly, in Figure 5B, band ④ with slower migration than band ③ emerged at a 1:2 ratio, and its color deepened at a 1:4 ratio, demonstrating that a new substance of a higher molecular weight was produced. However, no corresponding peak was observed in Figure 4B. For another intramolecular tandem stacked dimer GGA8 (Figure 5C), adding a small amount of RHAU (1:1) resulted in a wide and slow-migrating band ② belonging to the GGA8–RHAU complex, which was of light color. In the meantime, band ① of the GGA8 dimer was still obvious, meaning that the binding of GGA8 to RHAU was not strong and the amount of the dimer–RHAU complex was small. However, with increasing RHAU, the color of band ① gradually became lighter, while the color of band ②deepened, representing that more complex was produced. When the G4:RHAU ratio reached 1:4, band ① disappeared, and band ② showed the darkest color, which was totally in line with the changes in peak 1 and peaks 2/3/4 in Figure 4C.

The native-PAGE results of 93del, T30177, and T30695 were similar to each other (Figure 5D–F). When RHAU was added, they all produced slow-migrating bands ② belonging to dimer–RHAU complexes. As the amount of RHAU increased, the color of bands ① of dimers gradually becomes lighter while the color of bands ② of complexes deepened, representing the formation of increasing complexes, which were in agreement with SEC results. When the G4:RHAU ratio was 1:2, bands ① of these three dimers disappeared, and only bands ② of dimer–RHAU complexes were left. Moreover, at ratios of 1:2 and 1:4, bands ② of the complexes remained the same as those at a 1:1 ratio, demonstrating that the interactions of 93del, T30177, and T30695 with RHAU reached saturation at a 1:2 ratio, which was in accord with SEC results. When dimer–RHAU interactions reached saturation, there were still small peaks of remaining free dimers in SEC chromatograms, but no corresponding electrophoretic bands existed, which was also due to the rather small amounts of remaining dimers. Additionally, peaks 3 of 93del and T30695 in SEC had no corresponding electrophoretic bands. These findigs also pointed out that the SEC method is more sensitive to the detection of trace substances and is capable of supplementing details that are easily missed by other methods.

Compared to bands ① of 93del, T30177, and T30695, which disappeared at a 1:2 ratio, band ① of GGA8 disappeared at a 1:4 ratio, indicating that the interaction between GGA8 and RHAU was weaker than the interactions of these three G4s with RHAU, which was also consistent with chromatographic analysis.

These electrophoretic results fully confirmed the accuracy and reliability of SEC results, demonstrating that SEC can be effectively used in studies of G4 dimer–RHAU interactions.

## 3. Materials and Methods

### 3.1. Materials and Reagents

**Figure 6:**
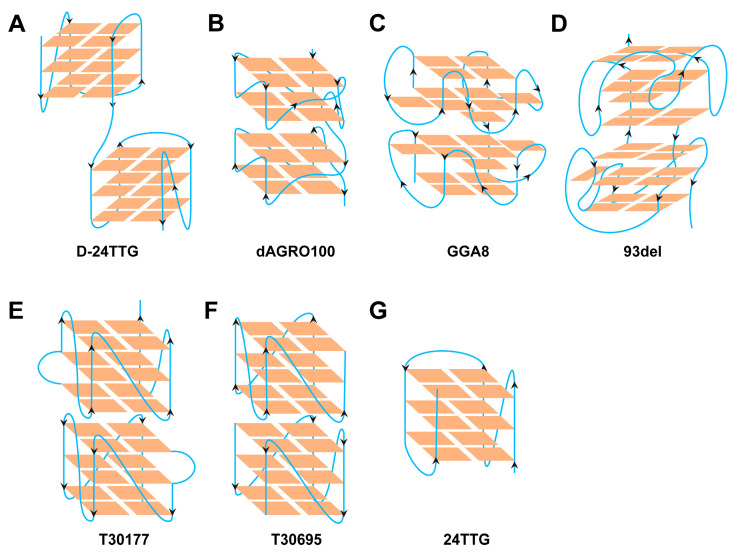
The structures of G4 dimers studied in this work.

The oligonucleotides (Table 1 and Figure 6) and the 55-amino-acid-RHAU peptide (N′-SMHPGHLKGREIGMWYAKKQGQKNKEAERQERAVVHMDERREEQIVQLLNSVQAK-C′) were all custom-synthesized by Sangon Biotech Co., Ltd. (Shanghai, China). The water used was ultrapure water. All of the other chemicals were of analytical reagent grade unless otherwise noted.

### 3.2. Sample Preparation

Single-stranded oligonucleotides were dissolved in Tris-HCl buffer (10 mM Tris-HCl, 100 mM KCl, pH 7.4) and quantified using UV–Vis absorption spectroscopy with the following extinction coefficients (ε_260 nm_, M^−1^ cm^−1^) for each nucleotide: A = 15,400, G = 11,500, C = 7400, and T = 8700. DNA solutions were heated in a 95 °C water bath for 5 min and then were cooled to room temperature naturally in order to prepare G4s. The cooled solutions were kept at 4 °C overnight to be used as stock solutions (100 μM), followed by being stored at −80 °C. RHAU was dissolved in ultrapure water and also stored at −80 °C. Before use, the stock solutions of RHAU and G4s were diluted to the required concentrations.

### 3.3. CD Experiments and UV–Vis Absorption Spectroscopy

CD measurements were performed using a Chirascan digital circular dichroism spectropolarimeter (Applied Photophysics Ltd., Leatherhead, UK) and a 1-mm-path-length quartz cuvette. The sampling interval was 0.5 s, and the slit width was 1 nm. Resulting measurements were the average of three repetitions between 220 nm and 330 nm at room temperature. CD spectra of the baseline and buffer were subtracted from these spectra of G4 solutions. All G4s for CD measurements were prepared at a concentration of 10 μM. For G4+RHAU mixtures, the ratios of G4:RHAU were set at 1:0, 1:1, 1:2, and 1:4. CD melting experiments were performed in the temperature range of 20–95 °C, with a heating rate of 1 °C/min, by monitoring the ellipticity at the characteristic wavelength of G4s.

UV–Vis absorptions were performed using a UH5300 UV-Vis spectrophotometer (Hitachi, Japan) and a 1-cm-path-length quartz cuvette. Absorbances were measured from 190 nm to 350 nm at room temperature. The UV–Vis spectrum of the background was subtracted from these spectra of G4 solutions. G4s for UV–Vis measurements were prepared at a concentration of 2 μM. For G4+RHAU mixtures, the ratios of G4:RHAU were set at 1:0, 1:1, 1:2, and 1:4.

### 3.4. SEC Conditions

SEC experiments were performed on an Agilent 1200 series high-performance liquid chromatograph (Agilent, Santa Clara, CA, USA) with a diode array detector. Separation was accomplished on a TSK_gel_ G2000SW_XL_ column (7.8 × 300 mm i.d.). The column temperature was maintained at 30 °C, and the detection wavelength was set at 260 nm for G4s and G4+RHAU mixtures. KH_2_PO_4_ buffer solution (50 mM, pH 7.0, adjusted by KOH) was used as a basic mobile phase for isocratic elution at a flow rate of 0.5 mL min^−1^. The final concentration of G4s was 10 μM, with an injection volume of 10 μL, and the ratios of G4:RHAU were set at 1:0, 1:1, 1:2, and 1:4.

### 3.5. Native-PAGE Experiments

Native-PAGE experiments were carried out on a Biorad PowerPac^TM^ HV apparatus (Bio-Rad, Hercules, CA, USA). G4s were prepared at a concentration of 50 μM in order to provide clear bands for analysis. For G4+RHAU mixtures, the ratios of G4:RHAU were set at 1:0, 1:1, 1:2, and 1:4. Electrophoresis was performed using 20% polyacrylamide gels containing 50 mM KCl and 1 × TBE buffer (80 mM Tris-borate, 2 mM EDTA, pH 8.3). G4s and G4+RHAU mixtures were run in 1 × TBE buffer supplemented with 50 mM KCl by using the following parameters: ice-water bath, voltage = 120 V, time = 2 h. Bands in the gels were visualized by UV shadowing.

## 4. Conclusions

Many parallel G4s have the ability and tendency to form multimers, and it is found that G4 multimers possess some properties that are better than those of G4 monomers, such as a better applicational performance. As the most common type of multimer, the G4 dimer is the main existing form of some G4 sequences that is relatively easy to acquire. Currently, dimers are used as the representative of G4 multimers in many studies [26,29,30,50,51,52], which can provide a powerful reference for the study of multimers. In this work, six different G4 dimers were selected, and their interactions with RHAU were investigated by evaluating changes in SEC retention behaviors. It was found that RHAU has weak interactions with the intramolecular hybrid tandem unstacked dimer formed by D-24TTG, while no interactions were observed between RHAU and the corresponding hybrid G4 monomer formed by 24TTG. This finding indicated that the hybrid tandem unstacked dimer produces some special sites different from that of the hybrid G4 monomer, which promotes the interaction with RHAU. Further analysis revealed that the interaction between RHAU and the Z-G4 dimer is much weaker than that between RHAU and common parallel dimers, which is due to the large terminal steric hindrance in the Z-G4 structure. This finding reminds us that the steric hindrance at terminal G-tetrads is still the main factor influencing the strength of interactions between RHAU and G4s. A comparison of 93del, T30177, and T30695 with GGA8 showed that RHAU interacts more strongly with these parallel intermolecular interlocked/stacked dimers than with the parallel intramolecular tandem stacked dimer, suggesting a possible recognition of RHAU to different types of G4 dimers. This work elucidates the interactions of RHAU with diverse G4 dimers, which is beneficial not only for the development and screening of specific ligands targeting G4 multimers but also for the analysis of G4 structural polymorphisms.

## Figures and Tables

**Table 1:** DNA sequences used in this work.

G4s	DNA Sequences (5′ to 3′)	Description	G4 Conformation
D-24TTG	AGGGTTAGGGTTAGGGTTAGGGTTAGGGTTAGGGTTAGGGTTAGG	Human telomere	Tandem unstacked hybrid dimer [27,37]
dAGRO100	TGGTGGTGGTGGTTGTGGTGGTGGTGTT	Derivative of aptamer AGRO100	Tandem stacked left-handed dimer [40]
GGA8	GGAGGAGGAGGAGGAGGAGGAGGA	GGA triplet repeats from eukaryotic genomes	Tandem stacked parallel dimer [38]
93del	GGGGTGGGAGGAGGGT	Inhibitors of HIV-1 integrase	Interlocked bimolecular parallel dimer [11]
T30177	GTGGTGGGTGGGTGGGT	Inhibitors of HIV-1 integrase	Stacked parallel dimer with a bulge [12]
T30695	GGGTGGGTGGGTGGGT	Inhibitors of HIV integrase	Stacked parallel dimer [39]
24TTG	TTGGGTTAGGGTTAGGGTTAGGGA	Human telomere	Hybrid monomer [46]

Note: The red font points out the position of the bulge in T30177.

**Table 2:** The assignment of the chromatographic peaks in Figure 4.

G4s	Molecular Weight (M_w_)	Peaks	t_R_/min	Corresponding Substances
D-24TTG	14,682.53	Peak 1	13.553	Tandem unstacked hybrid dimer
		Peak 2	12.145	Incompletely folded sequence
		Peak 3	13.225	Dimer–RHAU complex
dAGRO100	8934.77	Peak 1	15.060	Tandem stacked left-handed dimer
		Peak 2	14.552	Tandem unstacked left-handed dimer
		Peak 3	13.946	Incompletely folded sequence
		Peak 4	14.420	Dimer–RHAU complex
GGA8	7790.05	Peak 1	15.207	Tandem stacked parallel dimer
		Peak 2	14.525	Dimer–RHAU complex (intermediate I)
		Peak 3	14.450	Dimer–RHAU complex (intermediate II)
		Peak 4	14.300	Dimer–RHAU complex (stable state)
93del	5202.35	Peak 1	14.850	Interlocked bimolecular parallel dimer
		Peak 2	11.0–13.8	Structures of higher molecular weight
		Peak 3	14.086	Dimer–RHAU complex (intermediate)
		Peak 4	13.936	Dimer–RHAU complex (stable state)
T30177	5448.53	Peak 1	14.777	Stacked parallel dimer with a bulge
		Peak 2	14.075	Dimer–RHAU complex (intermediate)
		Peak 3	13.947	Dimer–RHAU complex (stable state)
T30695	5184.33	Peak 1	14.829	Stacked parallel dimer
		Peak 2	14.197	Stacked parallel trimer
		Peak 3	11.0–14.1	Stacked parallel multimer
		Peak 4	14.272	Dimer–RHAU complex (intermediate)
		Peak 5	14.051	Dimer–RHAU complex (stable state)
		Peak 6	15.050	Unknown impurity

## Data Availability

Data are contained within the article.

## Supplementary Materials

The following supporting information can be downloaded at https://www.mdpi.com/article/10.3390/molecules29245915/s1: Figure S1. CD melting spectra of G4 dimers without and with RHAU. (A) D-24TTG; (B) dAGRO100; (C) GGA8; (D) 93del; (E) T30177; (F) T30695. G4 concentration was fixed at 10 μM, and the molar ratios of G4:RHAU were 1:2. Table S1. Thermal stabilization of G4 dimers in the absence and presence of RHAU. Figure S2. Docking simulation diagrams. (A) 24TTG binds to RHAU peptide; (B) D-24TTG binds to RHAU peptide. Cyan represents the RHAU peptide, blue reparents the G4 skeleton, and purple represents the G base.
