# Supplementary material for: Exploring the Interactions Between RHAU Peptide and G-Quadruplex Dimers Based on Chromatographic Retention Behaviors
Source: Molecules. 2024 Dec 14;29(24):5915. doi: 10.3390/molecules29245915 (PMC11676799; doi:10.3390/molecules29245915)
Supplement: Supplementary file 1 [file molecules-29-05915-s001.zip › molecules-3305702-supplementary.pdf]

Supporting information

Exploring the interactions between RHAU peptide and G-quadruplex dimers based on chromatographic retention behaviors

Ju Wang [1], Jun-qin Qiao [1,*], Chao Liang [2], Xue-wen Guo [1], Meng-ying Zhang [2,*], Wei-juan Zheng [3], and Hong-zhen Lian [1,*]

1 State Key Laboratory of Analytical Chemistry for Life Science, School of Chemistry & Chemical Engineering and Center of Materials Analysis, Nanjing University, 163 Xianlin Avenue, Nanjing 210023, China

2 Nanjing Zhulu Pharmaceutical Technology Co., Ltd., 28 Kexin Road, Nanjing 211500, China

3 State Key Laboratory of Pharmaceutical Biotechnology, School of Life Sciences, Nanjing University, 163 Xianlin Avenue, Nanjing 210023, China

* Correspondence: qiaojunqin@nju.edu.cn (J.Q.Q.); zhangmyly1234@sina.com (M.Y.Z.); hzlian@nju.edu.cn (H.Z.L.)

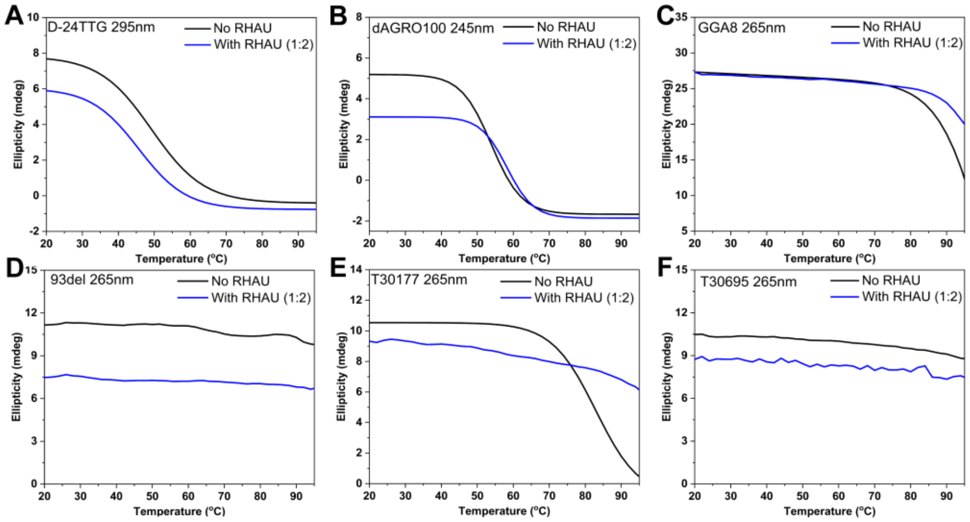

Figure S1. CD melting spectra of G4 dimers without and with RHAU. (A) D-24TTG; (B) dAGRO100; (C) GGA8; (D) 93del; (E) T30177; (F) T30695. G4 concentration was fixed at 10 μM, and the molar ratios of G4:RHAU were 1:2.

Table S1. Thermal stabilization of G4 dimers in the absence and presence of RHAU.

Sequences	Detection Wavelength (nm)	T_m (°C)		ΔT_m (°C)
		G4	G4+RHAU	
D-24TTG	295	49.3	45.7	-3.8
dAGRO100	245	53.9	58.2	4.3
GGA8	265	100.8	>100.8	/
93del	265	/	/	/
T30177	265	82.9	>82.9	/
T30695	265	/	/	/

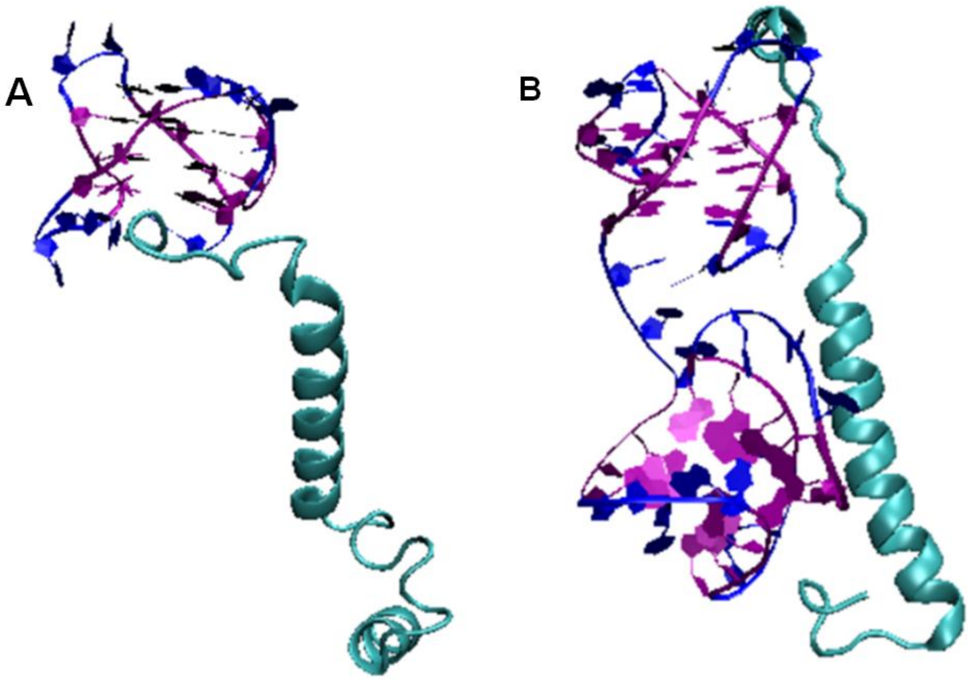

Figure S2. Docking simulation diagrams. (A) 24TTG binds to RHAU peptide; (B) D-24TTG binds to RHAU peptide. Cyan represents the RHAU peptide, blue reparents the G4 skeleton, and purple represents the G base.
